# Hemostasis Using Prothrombin Complex Concentrate in Patients Undergoing Cardiac Surgery: Systematic Review with Meta-Analysis

**DOI:** 10.21470/1678-9741-2023-0076

**Published:** 2024-02-26

**Authors:** Jun-Ping Li, Yan Li, Bing Li, Chang-He Bian, Feng Zhao

**Affiliations:** 1 Medical Oncology, Zibo Munícípal Hospítal, Zibo, Shandong, People’s Republic of Chína; 2 Department of Blood Transfusion, Zíbo Municípal Hospital, Zíbo, Shandong, People's Republic of Chína; 3 Thoracíc and Cardíovascular Surgery, Zíbo Municipal Hospital, Zibo, Shandong, People’s Republic of China

**Keywords:** Cardiac Surgery, Prothrombin Complex Concentrate, Hemorrhage, Mortality, Myocardial Infarction, Meta-Analysís, Systematic Review

## Abstract

**Objective:**

The purpose of present study was to comprehensívely explore the efficacy and safety of prothrombín complex concentrate (PCC) to treat massíve bleedíng in patíents undergoing cardiac surgery.

**Methods:**

PubMed®, Embase, and Cochrane Líbrary databases were searched for studíes ínvestigating PCC administratíon duríng cardiac surgery published before September 10, 2022. Mean dífference (MD) wíth 95% confidence interval (CI) was applíed to analyze continuous data, and dichotomous data were analyzed as risk ratio (RR) with 95% CI.

**Results:**

Twelve studies were included in the meta-analysis. Compared with other non-PCC treatment regimens, PCC was not assocíated with elevated mortality (RR=1.18, 95% CI=0.86–1.60, *P*=0.30, I^2^=0%), shorter hospital stay (MD=-2.17 days; 95% CI=-5.62–1.28, *P*=0.22, I^2^=91%), reduced total thoracic drainage (MD=-67.94 ml, 95% CI=-239.52–103.65, *P*=0.44, I^2^=91%), thromboembolíc events (RR=1.10, 95% CI=0.74–1.65, *P*=0.63, I^2^=39%), increase ín atríal fibríllatíon events (RR=0.73, 95% CI=0.52–1.05, *P*=0.24, I^2^=29%), and myocardial infarction (RR=1.10, 95% CI=0.80–1.51, *P*=0.57, I^2^=81%). However, PCC use was associated with reduced intensive care unit length of stay (MD=-0.81 days, 95% CI=-1.48– -0.13, *P*=0.02, I^2^=0%), bleeding (MD=-248.67 ml, 95% CI=-465.36– -31.97, *P*=0.02, I^2^=84%), and intra-aortic balloon pump/extracorporeal membrane oxygenation (RR=0.65, 95% CI=0.42–0.996, *P*=0.05, I^2^=0%) when compared with non-PCC treatment regimens.

**Conclusion:**

The use of PCC in cardiac surgery did not correlate with mortality, length of hospítal stay, thoracic drainage, atríal fibríllatíon, myocardíal ínfarction, and thromboembolíc events. However, PCC sígnificantly improved postoperatíve intensíve care unít length of stay, bleedíng, and intra-aortic balloon pump/ extracorporeal membrane oxygenation outcomes ín patients undergoing cardíac surgery.

## INTRODUCTION

Prothrombin complex concentrate (PCC) is a mixture of various coagulation factors and other plasma proteins extracted from the plasma supernatant after precipitation. Although originally used to treat hemophilia, PCC is now more commonly recommended to reverse massive bleeding induced by anticoagulants such as warfarin and the newer direct oral anticoagulants^[1,2]^.

The annual incidence of warfarin-related major bleeding is approximately 1 to 3%, and the case fatality rate is approximately 11%^[3]^. Patients undergoing cardiac surgery and cardiopulmonary bypass (CPB) frequently experience bleeding and coagulation dysfunction^[4,5]^, necessitating massive blood transfusions, as well as significantly increased mortality. Therefore, active and effective bleeding management critically impacts the prognosis of patients undergoing cardiac surgery. PCC is considered a potentially effective alternative to fresh frozen plasma (FFP) in patients experiencing massive bleeding after cardiac surgery^[6,7,8,9]^. Drug regimens play a positive role in surgical hemostasis, and common drugs promoting coagulation system functions include PCC, FFP, and recombinant factor VIIa (rFVIIa). Studies have shown that although FFP can be used to treat hemostasis, it could significantly increase vascular volume and lead to decompensated heart failure or transfusion-related lung injury. Therefore, FFP is rarely used for anticoagulant reversal in patients with atrial fibrillation, cardiovascular disease, and ventricular dysfunction^[10]^. In addition, the use of rFVIIa is reportedly associated with an increased risk of thrombotic events^[11]^. PCC administration was more effective than FFP in patients who experienced significant bleeding during cardiac surgery, reducing perioperative blood transfusions^[12]^. In addition, studies have found that a low PCC dosage has been shown to significantly reduce bleeding post-CPB^[9]^. Furthermore, PCC is superior to FFP to treat bleeding in patients presenting the need for emergency or invasive warfarin reversal^[13]^. Although PCC exhibits a superior ability to control massive bleeding, the effectiveness and safety of PCC need to be further clarified^[14]^. Therefore, the purpose of the present study was to comprehensively explore the efficacy and safety of PCC to treat massive bleeding in patients undergoing cardiac surgery by using more recently conducted or published studies.

## METHODS

The guidelines from the Preferred Reporting Items for Systematic Review and Meta-Analyses (or PRISMA) statement were employed for this study.

### Search Strategy

All studies investigating PCC use during cardiac surgery were obtained by searching the PubMed®, Embase, and Cochrane Library databases for articles published before September 10, 2022. The search terms were as follows: prothrombin complex concentrate, factor IX, factor 9, autoprothrombin II, Christmas factor, plasma thromboplastin component, cardiac surgical procedures, thoracic surgery, heart surgery, and cardiac surgery. Detailed search strategies were shown in Supplementary Method 1. Two reviewers (JPL and YL) independently assessed abstracts and potentially eligible articles identified during the literature selection, and discrepancies were resolved through discussion. The third reviewer (FZ) was consulted in the case of any disagreements.

### Inclusion and Exclusion Criteria

Inclusion criteria were as follows: (a) population: adult patients undergoing cardiac surgery; (b) intervention: three- or four-factor PCC; (c) control: non-PCC patients, including FFP, rFVIIa, or no treatment; (d) outcomes: mortality, length of hospital stay, intensive care unit (ICU) stay, blood loss, thoracic drainage, thromboembolic events, and intra-aortic balloon pump (IABP)/ extracorporeal membrane oxygenation (ECMO); the mortality rate was the all-cause mortality rate within 90 days after surgery; if there were multiple time points, the longest time node data within 90 days were selected; except for the length of hospital stay and length of ICU stay, it was 24 hours after surgery; (e) study type: randomized controlled trial (RCT), cohort study, or case-control study.

Exclusion criteria were as follows: repetitive studies, unavailability of data on contacting authors, cardiac surgery without thoracotomy, case reports, letters, and meeting abstracts.

### Data Extraction

Based on inclusion and exclusion criteria, two authors independently selected studies for inclusion by reading abstracts and full-text articles. In the event of any disagreement due to inconsistent understanding, a consensus was reached by arbitration and discussion with a third investigator. The following information was extracted from all trials: first author, age, sex, race, sample, body mass index, previous history of heart disease, type of surgery, PCC, and non-PCC.

### Quality Assessment

Regarding the quality of the included studies, two reviewers (JPL and YL) independently assessed the quality of RCT according to criteria reported in the Cochrane Handbook^[15]^. The included studies were assessed based on the following items scored as high, low, and unclear risks: random sequence generation, allocation concealment, blinding of participants and personnel, blinding of outcome assessors, incomplete outcome data, selective reporting, and other sources of bias. The methodological quality of included cohort studies or case-control studies were assessed using the Newcastle-Ottawa Scale (NOS), independently evaluated by two commentators. Studies that achieved six or more stars on the modified NOS were considered high quality. Any disagreement was resolved by discussion and consultation with a third author (FZ) if necessary.

### Statistical Analysis

Mean difference (MD) with 95% confidence intervals (CIs) was applied to analyze continuous data, and dichotomous data were analyzed as risk ratios (RRs) with 95% CIs. The I^2^ statistics were used to assess the heterogeneity of each analysis. I^2^ was calculated from basic data to represent the size of heterogeneity. A value of 0% represents no heterogeneity, and larger values suggest increased heterogeneity. A fixed-effects model was employed when I2 < 40%, whereas a random-effects model was used when I^2^ ≥ 40%. All statistical methods were performed according to the Cochrane Handbook^[15]^, and all statistical analyses were performed using RevMan 5.4.1.

## RESULTS

### Characteristics of Included Trials

**Fig. 1:**
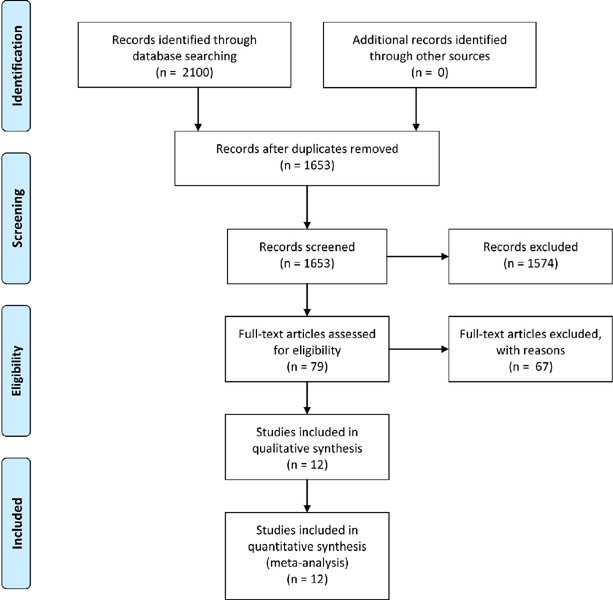
Preferred Reporting Items for Systematic Review and Meta-Analyses (or PRISMA) flow diagram.

Our systematic literature search identified 2,100 potential publications (Figure 1). Based on inclusion and exclusion criteria, we obtained quantitative data for the present meta-analysis by reading all titles, abstracts, and full-text evaluations. Subsequently, 12 studies^[6,7,8,16,17,18,19,20,21,22,23,24]^ assessing 1,799 participants were included (Table 1).

**Table 1:** Basic information regarding included studies.

Study, year	Age (years) (PCC/Non-PCC)	Female (PCC/Non-PCC)	Sample (PCC/Non-PCC)	BMI (PCC/Non-PCC)	Types of cardiac surgery	PCC group	Non-PCC group
Biancari, 2019	65.9 (6.7)/65.3 (9.3)	14-nov.	101/101	27.4 (4.3)/27.1 (3.9)	CABG	PCC: initial dose was 1,000 IU (2,000-3,000 IU)	Fresh frozen plasma with non-PCC
Bradford, 2015	68/69	3-mai.	41/27	NA	CABG	PCC: 500 units, every 30 min., maximum dose of 25 units/kg	Non-PCC
Cappabianca, 2016	69.7 (10.6)/69.2 (11.6)	91/91	225/225	24 (4.9)/25 (4.7)	CABG, valve surgery, and proximal aortic procedures	PCC: 500 IU	Non-PCC
Fitzgerald, 2018	61 (46-70)/60 (50-69)	45/40	117/117	NA	Valve or isolated CABG	PCC: 15-25 lU/kg in 1000 IU increments	Frozen plasma with non-PCC
Green, 2020	69 (63-73)/66 (57-74)	9-set.	21/21	27 (6)/29 (5)	Valve only, major aortic valve only, CABG plus valve, complex/combined procedure	<60 kg: 500 IU; 61-90 kg: 1000 IU; and > 90 kg: 1500 IU	Fresh frozen plasma with non-PCC
Harper, 2018	60.9 (17.4)/58.5 (19.7)	17/18	53/53	NA	Mechanical circulatory support, valve transplant, aorta resection plus CABG/valve (s), aorta resection, CABG plus valve (s), congenital/ conduit, CABG, pericardiectomy	Factor IX complex	rFVIIa
Harris, 2020	65.7 (59-77)/66.9 (59-73)	mai.-19	19/60	28.7 (24.5-34.3)/29.7 (25.1-33.3)	Isolated CABG, isolated valve	4-factor PCC: 11.5 (5.3-39.3) units/kg	Non-4-factor PCC
Karkouti, 2020	66 (50-73)/67 (55-74)	14/14	54/47	23.6 (4.5)/23.1 (4.7)	Cardiac surgery	PCC: 1500 IU for patients weighing ≤ 60 kg and 2000 IU for patients weighing > 60 kg	Frozen plasma with non-PCC
Ortmann, 2014	61 (13)/62 (13)	19/18	45/55	28.8(6.1)/27.3(5.0)	Complex cardiac surgery	PCC:15 IU/kg to the nearest 250 IU vial	Fresh frozen plasma with non-PCC
Tanaka, 2013	55.5 (16.6)/57.8 (12.6)	15/30	50/100	NA	Valve, aortic, transplant or ventricular assist device implantation	3-factor PCC: 25 lU/kg, Bebulin or Profilnine upon availability	rFVIIa
Zweng, 2018	66.9 (12.18)/69.4 (10.5)	25/34	80/80	NA	CABG, valve, other	The amount of PCC: 500 to 9000 IU	Fresh frozen plasma with non-PCC
Alyson, 2021	65 (57-73) /69 (58-78)	21-set.	61/46	25.2 (22.5-29.1)/25 (23-28)	CABG, multivalve procedure, single-valve procedure, CABG and valve, aortic procedure, aortic dissection, ventricular septal defect repair	4-factor PCC	rFVIIa

BMI=body mass index; CABG=coronary artery bypass grafting; IU=international units; NA=not available; PCC=prothrombin complex concentrate; rFVIIa=recombinant factor Vlla

### Quality Assessment

Among the included studies, there were two RCTs, two cohort studies, and the rest eight studies were case-control studies. Table 2 shows the risk of bias according to criteria reported in the Cochrane Handbook for RCTs, Table 3 shows the NOS scoring system for cohort studies, and Table 4 shows the NOS scoring system for case-control studies. All the included studies were considered high quality.

**Table 2:** Quality assessment of randomized controlled trials.

Author	Year	Random sequence generation	Allocation concealment	Blinding of participants and personnel	Blinding of outcome assessors	Incomplete outcome data	Selective reporting	Other
Green	2020	Low	Low	Low	Low	Low	Low	Unclear
Karkouti	2020	Low	Low	Low	Low	Low	Low	Unclear

**Table 3:** Quality assessment of cohort studies using Newcastle-Ottawa Scale.

Author	Year	Selection				Comparability		Outcomes			Total
		Representativeness of the exposed cohort	Selection of the non-exposed cohort	Ascertainment of exposure	Demonstration that outcome of interest was not present at start of study	Comparability of cohorts on the basis of the design or analysis		Assessment of outcome	Was follow-up long enough for outcomes to occur?	Adequacy of follow-up of cohorts	
Biancari	2019	1	1	1	1	1	1	1	1	1	9
Ortmann	2014	1	1	1	1	0	1	1	1	1	8

**Table 4:** Quality assessment of case-control studies using Newcastle-Ottawa Scale.

Author	Year	Selection				Comparability		Outcomes			Total
		Is the case definition adequate?	Representativeness of the cases	Selection of controls	Definition of controls	Comparability of cases and controls on the basis of the design or analysis		Ascertainment of exposure	Same method of ascertainment for cases and controls	Non-response rate	
Bradford	2015	1	1	1	0	1	1	1	1	1	8
Cappabianca	2016	1	1	1	1	0	1	1	1	1	8
Fitzgerald	2018	1	1	1	1	1	1	1	1	1	9
Harper	2018	1	1	1	0	1	1	1	1	1	8
Harris	2020	1	1	1	1	1	0	1	1	1	8
Tanaka	2013	1	1	1	0	1	0	1	1	1	7
Zweng	2018	1	1	1	1	0	1	1	1	1	8
Alyson	2021	1	1	0	1	1	0	1	1	1	7

### Result of Meta-Analysis

#### Mortality

**Fig. 2:**
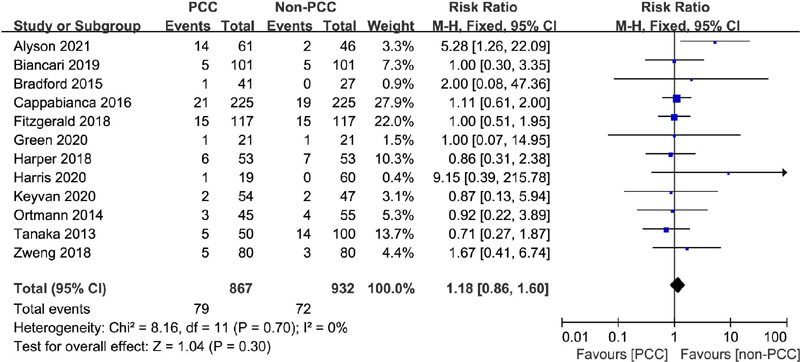
Comparison of mortality between patients treated with prothrombin complex concentrate (PCC) and those not treated with PCC. CI=confidence interval.

Mortality data were available for all 12 included studies^[6,7,8,16,17,18,19,20,21,22,23,24]^, with a total of 1,799 patients. Overall, death occurred in 79 of 867 patients in the PCC group and 72 of 932 patients in the non-PCC group. Accordingly, PCC use was not associated with increased mortality in any patient group (RR=1.18, 95% CI=0.86–1.60, *P*=0.30, I^2^=0%) (Figure 2).

#### Bleeding

**Fig. 3:**
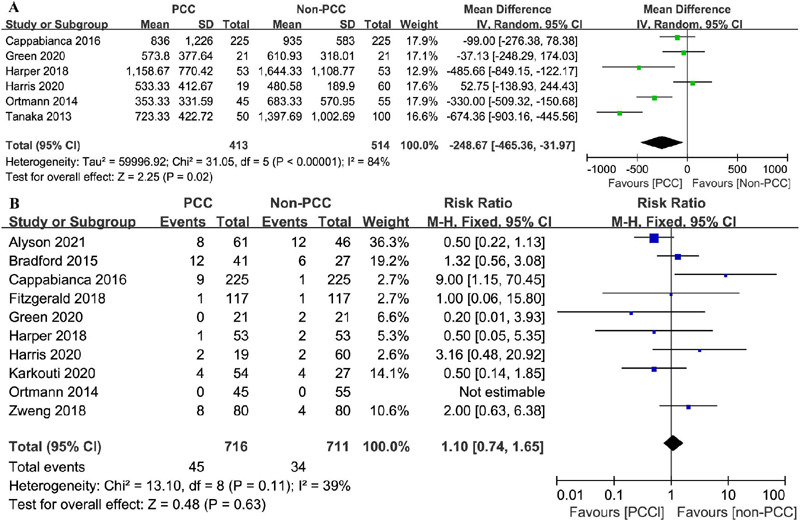
Comparison of bleeding and thromboembolic events between patients treated with prothrombin complex concentrate (PCC) and those not treated with PCC. A) Bleeding; B) thromboembolic events. CI=confidence interval; SD=standard deviation.

Blood loss data were available for six studies involving 927 patients undergoing cardiac surgery^[6,8,18,19,20,23]^. Patients who received PCC experienced an average blood loss of 353–1159 ml, whereas those in the non-PCC group presented an average blood loss of 480–1644 ml. The total blood loss in the PCC group was significantly decreased (MD=-248.67 ml, 95% CI=-465.36– -31.97, *P*=0.02, I^2^=84%) (Figure 3A).

#### Thromboembolic Events

Data on thromboembolic events were recorded in 10 studies^[6,7,8,17,18,19,20,21,22,24]^. Thromboembolic events occurred in 45 of 716 patients in the PCC group and 34 of 771 patients in the non-PCC group. PCC use was not associated with thromboembolic events (RR=1.10, 95% CI=0.74–1.65, *P*=0.63, I^2^=39%) (Figure 3B).

### Intra-aortic Balloon Pump/Extracorporeal Membrane Oxygenation

**Fig. 4:**
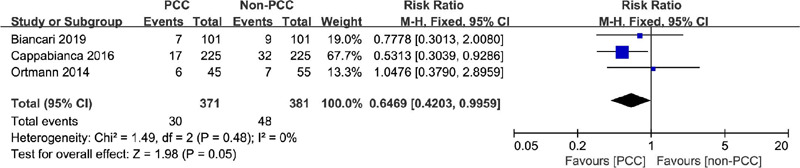
Comparison of intra-aortic balloon pump/extracorporeal membrane oxygenation between patients treated with prothrombin complex concentrate (PCC) and those not treated with PCC. CI=confidence interval.

The incidence of IABP/ECMO was recorded in three studies examining 752 patients^[6,8,16]^. IABP/ECMO was performed in 30 of 371 patients in the PCC group and 48 of 381 patients in the non-PCC group. In all patient groups, the use of PCC slightly reduced IABP/ECMO events (RR=0.65, 95% CI=0.42–0.996, *P*=0.05, I^2^=0%) (Figure 4).

#### Atrial Fibrillation

**Fig. 5:**
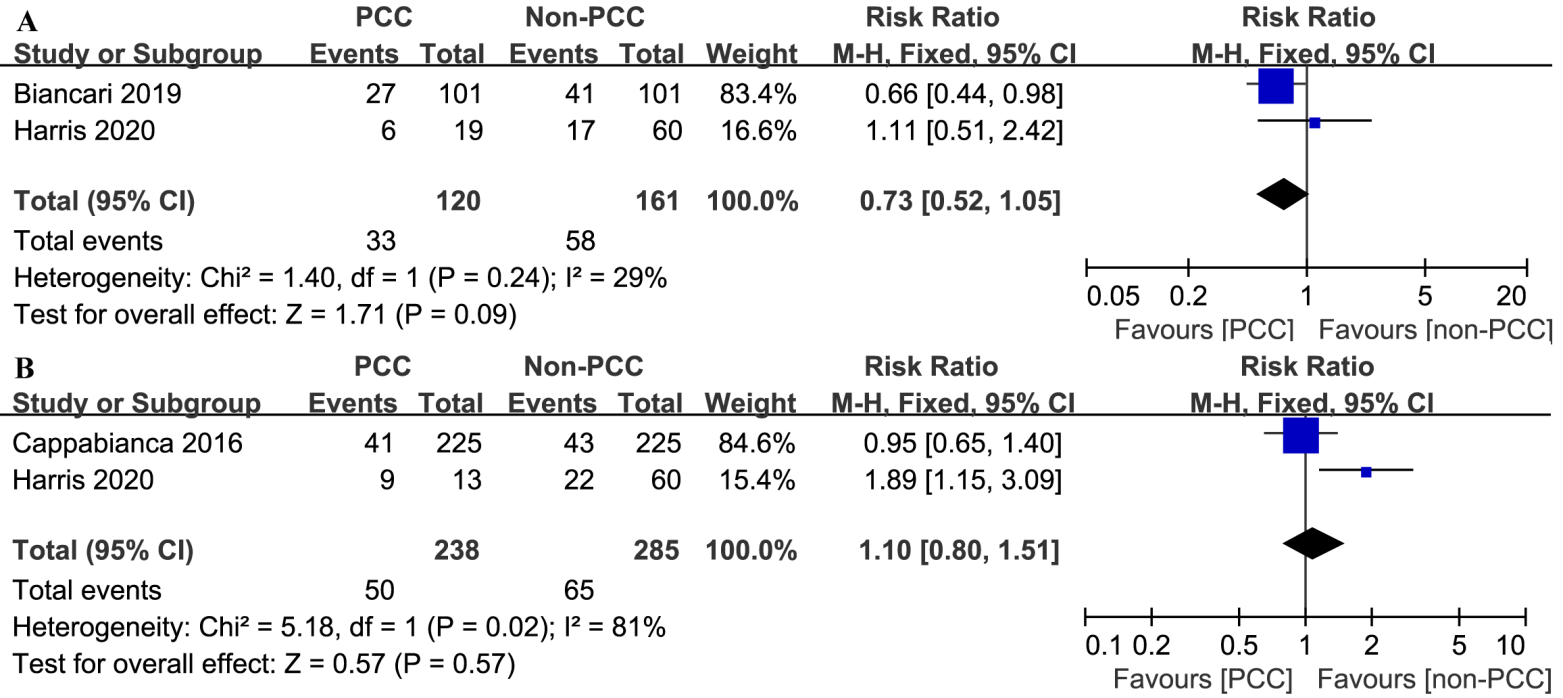
Comparison of atrial fibrillation and myocardial infarction between patients treated with prothrombin complex concentrate (PCC) and those not treated with PCC. A) Atrial fibrillation; B) myocardial infarction. CI=confidence interval.

Data on the occurrence of atrial fibrillation was recorded in two included studies^[16,20]^, with 281 patients in total. Overall, 33 events in 120 patients and 58 events in 161 patients were documented in PCC and non-PCC groups, respectively. PCC use was not associated with an increase in atrial fibrillation events in any patient group (RR=0.73, 95% CI=0.52–1.05, *P*=0.24, I^2^=29%) (Figure 5A).

#### Myocardial Infarction

Only two studies reported the incidence of myocardial infarction^[8,22]^. Among 238 patients in the PCC group, 50 presented with myocardial infarction, and among 285 patients in the non-PCC group, 65 exhibited myocardial infarction (RR=1.10, 95% CI=0.80–1.51, *P*=0.57, I^2^=81%) (Figure 5B).

#### Thoracic Drainage

**Fig. 6:**
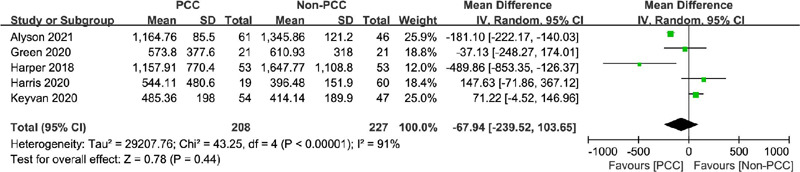
Comparison of thoracic drainage between patients treated with prothrombin complex concentrate (PCC ) and those not treated with PCC. CI=confidence interval; SD=standard deviation.

Thoracic drainage was reported in five studies^[18,19,20,21,22]^ evaluating 435 patients who underwent cardiac surgery. The mean thoracic drainage was 485–1165 ml in the PCC group and 396–1648 ml in the non-PCC group, with the total thoracic drainage significantly reduced in the PCC group (MD=-67.94 ml, 95% CI=-239.52–103.65, *P*=0.44, I^2^=91%) (Figure 6).

#### Hospital Length of Stay

**Fig. 7:**
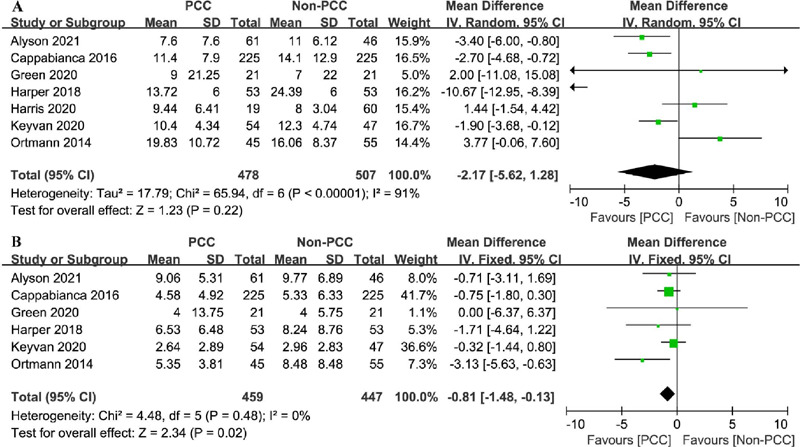
Comparison of hospital length of stay and intensive care unit (ICU) length of stay between patients treated with prothrombin complex concentrate (PCC) and those not treated with PCC. A) Hospital length of stay; B) ICU length of stay. CI=confidence interval; SD=standard deviation.

In total, 985 patients were examined in seven studies^[6,8,18,19,20,21,22]^, presenting a mean hospital stay of 7.6–19.8 days and 7–24.4 days in the PCC and non-PCC groups, respectively. PCC use was not associated with a shorter hospital stay (MD=-2.17 days; 95% CI=-5.62–1.28, *P*=0.22, I^2^=91%) (Figure 7A).

### Intensive Care Unit Length of Stay

A total of 906 patients were evaluated in six studies^[6,8,18,19,21,22]^. PCC use was associated with a shorter ICU stay (MD=-0.81 days, 95% CI=-1.48– -0.13, *P*=0.02, I^2^=0%) (Figure 7B).

#### Publication Bias

All funnel plots showed symmetry, and no publication bias was found in any outcome examined.

## DISCUSSION

In this meta-analysis, our findings revealed that PCC use was associated with reduced bleeding, IABP/ECMO, and ICU length of stay. In addition, PCC use did not increase mortality, thromboembolic events, atrial fibrillation, myocardial infarction, thoracic drainage, and hospital length of stay.

During aortic balloon counterpulsation, an inflatable balloon contracts and compresses the airbag, which decreases cardiac contractions, increases the cardiac ejection burden, and enhances coronary blood flow during diastole, thereby improving the blood supply to coronary arteries and reducing the cardiac backload^[25]^. IABP can effectively enhance myocardial blood supply and reduce oxygen consumption. In clinical settings, IABP is used to treat myocardial infarction, cardiogenic shock, and other serious coronary heart diseases or for the prevention and support of cardiac interventional surgery^[26]^. ECMO is primarily used to provide continuous external respiration and circulation in patients with severe cardiopulmonary failure for maintenance of life^[27]^. ECMO comprises a membrane lung (artificial lung) and blood pump (artificial heart), which can provide long-term cardiopulmonary support for patients with severe cardiopulmonary failure and afford the critical time required to rescue critically ill patients. No correlation was observed between PCC use and lung reperfusion injury; hence, it was speculated that ECMO in the present study was mainly used for cardiac treatment and could reduce IABP/ ECMO. As blood loss data included in the present study did not specify the specific site of blood loss, it was preliminarily discussed and predicted that the use of PCC might play a role in reducing cardiac blood loss. The analysis showed that PCC use was not associated with a reduction in myocardial infarction and atrial fibrillation, probably due to insufficient data and sample size in both areas. In conclusion, the use of PCC may have a beneficial effect on specific parts of the heart during the treatment of blood loss, and PCC can be used to treat severe perioperative bleeding during cardiac surgery. It is also possible that heterogeneity in several studies influenced the observed results.

PCC can effectively reduce hematoma formation in patients with trauma and quickly reverse the effect of vitamin K antagonist, which has greater advantages than FFP^[28]^. During initial resuscitation, combined with thromboelastography results, PCC combined with cryoprecipitation or human fibrinogen concentrate could effectively increase the coagulation time. rFVIIa can be considered when the best blood substitute treatment scheme, surgery, and anti-fibrinolysis have been comprehensively exploited, serious acidosis, hypothermia, and hypocalcemia have been corrected, and bleeding could not be effectively controlled (hematocrit > 24%, platelet > 50 × 109/L, and fibrinogen > 1.5–2.0 g/L). Studies have shown that, with the support of the abovementioned standards, the use of rFVIIa can effectively reduce mortality and the amount of blood transfusion required; however, it is necessary to be vigilant against the rFVIIa-induced arterial thrombosis^[29]^. It should be noted that high-quality studies supporting the use of rFVIIa as a first-line drug are seriously lacking. In addition, excessive PCC use should be avoided to prevent thrombosis. Studies have reported that three-factor complexes significantly increase the risk of thrombosis when compared with four-factor complexes. Therefore, real-time dynamic monitoring of coagulation-related indicators can help reduce the risk of thrombosis.

### Limitations

Although this study collected considerable research data and had a large sample size, limitations need to be addressed. First, different PCC types and doses can lead to distinct clinical effects, resulting in under- or over-description of actual effects. Second, not all included studies had strict inclusion or exclusion criteria for research subjects, resulting in population heterogeneity. Third, in some studies, the mean value was derived from the median and quartile, and the original research data were not recorded using the mean and variance, which may impact the accuracy of obtained results.

## CONCLUSION

Although the use of PCC in cardiac surgery did not correlate with mortality, length of hospital stay, thoracic drainage, atrial fibrillation, myocardial infarction and thromboembolic events, PCC significantly improved postoperative ICU length of stay, bleeding, and IABP/ECMO outcomes in patients undergoing cardiac surgery.

## Abbreviations, Acronyms & Symbols

BMI: Body mass index
CABG: Coronary artery bypass grafting
CI: Confidence interval
CPB: Cardiopulmonary bypass
ECMO: Extracorporeal membrane oxygenation
FFP: Fresh frozen plasma
IABP: Intra-aortic balloon pump
ICU: Intensive care unit
IU: International units
MD: Mean difference
NA: Not available
NOS: Newcastle-Ottawa Scale
PCC: Prothrombin complex concentrate
RCT: Randomized controlled trial
rFVIIa: Recombinant factor VIIa
RR: Risk ratio
SD: Standard deviation

## Supplementary Method 1 Search Strategy

**1. PubMed®, 1946 to September 10, 2022**

#1 prothrombin complex concentrate

#2 factor IX

#3 factor 9

#4 autoprothrombin II

#5 christmas factor

#6 plasma thromboplastin component

#7 #1 OR #2 OR #3 OR #4 OR #5 OR #6

#8 cardiac surgical procedures

#9 thoracic surgery

#10 heart surgery

#11 cardiac surgery

#12 #8 OR #9 OR #10 OR #11

#13 #7 AND #12

**2. Embase, <1974 to September 10, 2022>**

#1 ‘prothrombin complex concentrate’/exp

#2 factor IX:ti,ab

#3 factor 9:ti,ab

#4 autoprothrombin II:ti,ab

#5 plasma thromboplastin component:ti,ab

#6 Christmas factor:ti,ab

#7 #1 OR #2 OR #3 OR #4 OR #5 OR #6

#8 cardiac surgical procedures’/exp

#9 thoracic surgery:ti,ab

#10 heart surgery:ti,ab

#11 cardiac surgery:ti,ab

#12 #8 OR #9 OR #10 OR #11

#13 #7 AND #12

**3. Cochrane Central Register of Controlled Trials, <Issue 8 of 12, September 2022>**

#1 MeSH descriptor: [prothrombin complex concentrate] explode all trees

#2 (factor IX):ti,ab,kw

#3 (factor 9):ti,ab,kw

#4 (autoprothrombin II):ti,ab,kw

#5 (christmas factor):ti,ab,kw

#6 (plasma thromboplastin component):ti,ab,kw

#7 #1 OR #2 OR #3 OR #4 OR #5 OR #6

#8 MeSH descriptor: [cardiac surgical procedures] explode all trees

#9 (thoracic surgery):ti,ab,kw

#10 (heart surgery):ti,ab,kw

#11 (cardiac surgery):ti,ab,kw

#12 #8 OR #9 OR #10 OR #11

#13 #7 AND #12
